# RCAN1 Inhibits BACE2 Turnover by Attenuating Proteasome-Mediated BACE2 Degradation

**DOI:** 10.1155/2020/1920789

**Published:** 2020-06-05

**Authors:** Kaixin Qiu, Shuai Wang, Xin Wang, Fengting Wang, Yili Wu

**Affiliations:** ^1^Cheeloo College of Medicine, Shandong University, Wenhua West Road No. 44, Lixia District, Jinan, Shandong, China 250012; ^2^Shandong Collaborative Innovation Center for Diagnosis, Treatment and Behavioral Interventions, Institute of Mental Health, Jining Medical University, Hehua Road No. 133, Taibaihu New District, Jining, Shandong, China 272067; ^3^Shandong Key Laboratory of Behavioral Medicine, School of Mental Health, Jining Medical University, Hehua Road No. 133, Taibaihu New District, Jining, Shandong, China 272067

## Abstract

Amyloid-*β* protein (A*β*) is the main component of neuritic plaques, the pathological hallmark of Alzheimer's disease (AD). *β*-site APP cleaving enzyme 1 (BACE1) is a major *β*-secretase contributing to A*β* generation. *β*-site APP cleaving enzyme 2 (BACE2), the homolog of BACE1, is not only a *θ*-secretase but also a conditional *β*-secretase. Previous studies showed that regulator of calcineurin 1 (RCAN1) is markedly increased by AD and promotes BACE1 expression. However, the role of RCAN1 in BACE2 regulation remains elusive. Here, we showed that RCAN1 increases BACE2 protein levels. Moreover, RCAN1 inhibits the turnover of BACE2 protein. Furthermore, RCAN1 attenuates proteasome-mediated BACE2 degradation, but not lysosome-mediated BACE2 degradation. Taken together, our work indicates that RCAN1 inhibits BACE2 turnover by attenuating proteasome-mediated BACE2 degradation. It advances our understanding of BACE2 regulation and provides a potential mechanism of BACE2 dysregulation in AD.

## 1. Introduction

Alzheimer's disease (AD) is the most common neurodegenerative disease in the elderly. Neuritic plaques are the major pathological hallmark of AD, while amyloid-*β* protein (A*β*) is the main component of neuritic plaques [1]. A*β* is derived from amyloid-*β* precursor protein (APP) by sequential cleavage of *β*- and *γ*-secretase [2–4]. *β*-site APP cleaving enzyme 1 (BACE1) is the major *β*-secretase contributing to A*β* generation [2]. Sun et al. reported that *β*-site APP cleaving enzyme 2 (BACE2), the homolog of BACE1 cleaves APP at the Phe20 site to yield a C-terminal fragment (CTF) with 80 amino acids (CTF*θ* or C80), which is further cleaved by *γ*-secretase to release a truncated A*β* [3, 5, 6]. Thus, BACE2 is considered as a *θ*-secretase. Consistently, overexpression of BACE2 in primary neurons derived from Swedish mutant APP transgenic mice significantly reduces A*β* generation [6]. Moreover, BACE2 prevents neuronal apoptosis by cleaving a potassium channel at the surface of the neuronal plasma membrane [7].

However, growing evidence indicates that BACE2 may be implicated in the pathogenesis of AD. First, our recent study demonstrated that the binding of clusterin to the juxtamembrane helix (JH) of APP triggers BACE2-mediated *β*-cleavage of APP, indicating that BACE2 has comparable *β*-secretase activity to that of BACE1. Both BACE2 and clusterin are elevated in aged mouse brains, indicating that increased clusterin in the elderly may facilitate the *β*-secretase activity of BACE2, contributing to the pathogenesis of AD [8]. In addition, increased BACE2 expression and activity are detected in the neurons of AD brains [9]. Genetic data shows that BACE2 is associated with AD risk [10]. For example, the BACE2 haplotype associates with AD, while SNPs in BACE2 (e.g., rs2252576, rs2837990, and rs7281733) predispose to early onset of AD in Down syndrome. Recently, the association between SNPs in BACE2 and AD was reported in APOE *ε*4 noncarriers, which may be mediated by altered BACE2 expression-mediated A*β* generation and clearance [11].

Regulator of calcineurin 1 (RCAN1), also called Down syndrome critical region 1 (DSCR1), is highly expressed in the human brain and heart [12]. By alternative splicing and alternative promoter usage, two major transcripts are generated, transcript 1 and transcript 4, *RCAN1.1* and *RCAN1.4* [13–15]. Transcript 1 encodes two isoforms by alternative usage of two translational start sites, the long isoform of RCAN1.1 (RCAN1.1L) and the short isoform of RCAN1.1 (RCAN1.1S), which consist of 252 and 197 amino acids, respectively [16]. RCAN1.1L is the major isoform of RCAN1.1, which is upregulated in AD. However, RCAN1.1S is hard to be detected. Transcript 4 encodes RCAN1.4 with 197 amino acids. RCAN1.1L (hereinafter referred to as RCAN1) is highly expressed in the brains and is upregulated in AD brains. Increased RCAN1 plays a pivotal role in AD pathogenesis [15] including neuronal loss [17, 18], tau hyperphosphorylation [19, 20], and synaptic dysfunction [21, 22]. Previous study showed that RCAN1 significantly increases BACE1 expression, while BACE1 and BACE2 share an approximate 75% similarity of amino acids. However, the role of RCAN1 in BACE2 regulation remains elusive. In this study, we reported that RCAN1 increases BACE2 protein levels. Moreover, RCAN1 inhibits the turnover of BACE2 protein. Furthermore, RCAN1 attenuates proteasome-mediated BACE2 degradation, but not lysosome-mediated BACE2 degradation. Taken together, our work indicates that RCAN1 inhibits BACE2 turnover by attenuating proteasome-mediated BACE2 degradation, leading to the upregulation of BACE2. It advances our understanding of BACE2 regulation and provides a potential mechanism of BACE2 dysregulation in AD.

## 2. Materials and Methods

### 2.1. Cell Culture and Transfection

Human embryonic kidney HEK293 cells and HRNLM cells obtained from Dr. Weihong Song's lab were cultured in high-glucose DMEM containing 10% fetal bovine serum and 1% penicillin-streptomycin. HRNLM cells are derived from HEK293 cells, which stably overexpress RCAN1 with a C-terminal myc tag. All cells were maintained at 37°C with 5% CO_2_ in an incubator as described previously [16, 23, 24]. pBACE2-mycHis refers to pZ-BACE2mycHis in this study, which is constructed previously [25]. Transient transfection was performed by using the polyetherimide (PEI) method as described previously [26, 27]. Briefly, HEK293 and HRNLM cells were seeded 24 h prior to transfection. The regular culture medium was replaced with high-glucose DMEM without serum 1 h prior to transfection. 6 h after transfection, the medium was replaced with regular culture medium.

### 2.2. Pharmacological Treatments

HEK293 cells and HRNLM cells were transiently transfected with pBACE2-mycHis. 24 h after transfection, the cells were equally seeded into 6 cm culture dishes. 48 h after transfection, the cells were treated with different drugs, respectively. To measure the half-life of BACE2, 100 *μ*g/mL cycloheximide (CHX) was used to treat cells as described previously [16, 23, 24]. After treatment, the cells were harvested at 0, 2, 4, 8, and 12 h time points, respectively. The lysosomal inhibitor NH_4_Cl was applied to determine the involvement of the lysosome pathway in BACE2 degradation, while the proteasomal inhibitor N-carbobenzoxy-L-leucinyl-L-leucinyl-L-leucinal (MG-132) was applied to determine the involvement of proteasome pathway in BACE2 degradation, respectively [16, 23, 28–30]. MG-132 and NH_4_Cl were purchased from Sigma.

### 2.3. Western Blotting

Cells were lysed with RIPA-Doc buffer (Tris-HCl, 50 mM; NaCl, 150 mM; Triton X-100, 1%; deoxycholate, 1%; and SDS, 0.1%; supplemented with 1/100 protease inhibitors). Cell lysates were separated by 10% Tris-glycine SDS-PAGE gels and transferred to PVDF membranes. The membranes were blocked in 5% nonfat milk for 1 h, then incubated overnight at 4°C with anti-myc (9E10), anti-GFP, anti-RCAN1, and anti-*β*-actin (AC-15) antibodies, respectively [23]. The membranes were washed in TBST with 0.1% Tween-20 and incubated with HRP-labeled goat anti-mouse or HRP-labeled goat anti-rabbit antibodies at room temperature for 1 h. Anti-myc antibody 9E10 was obtained from Abcam. *β*-Actin antibody AC-15, HRP-labeled goat anti-rabbit antibody, and HRP-labeled goat anti-mouse antibody were obtained from ZSGB-BIO. Anti-GFP antibody was purchased from Proteintech Group. Anti-RCAN1 antibody was purchased from Sigma. The image was obtained by using FluorChem R imaging system.

### 2.4. Statistical Analysis

The protein expression was quantified by using ImageJ. Student's *t* test or two-way ANOVA was used for data analysis with three or more independent experiments. *P* < 0.05 was considered as a significant difference.

## 3. Results

### 3.1. RCAN1 Increases BACE2 Expression

**Figure 1:**
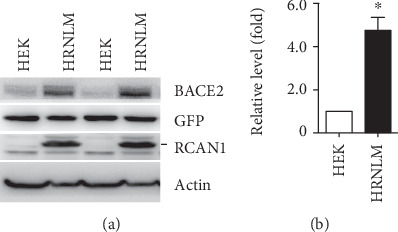
RCAN1 increases BACE2 expression. (a) HEK293 cells and HRNLM cells were cotransfected with plasmids pEGFP and pBACE2-mycHis. Cell lysates were resolved by 10% SDS-PAGE. BACE2 expression was detected by using 9E10 antibody. GFP was detected by GFP antibody. Anti-RCAN1 antibody was used to detect RCAN1. *β*-Actin was detected by AC-15 antibody. (b) Quantification of BACE2 levels. GFP levels were used to normalize the transfection efficiency. Values are mean ± SEM; *n* ≥ 3, ∗*P* < 0.05 by Student's *t* test.

To explore the effect of RCAN1 on BACE2 regulation, HEK293 cells and HRNLM cells (i.e., HEK293 cells stably overexpressing myc-tagged RCAN1) were cotransfected with plasmids pEGFP and pBACE2-mycHis at the ratio of 1 : 5. Exogenous GFP was used as a control for transfection efficiency in both cell lines. We found that the level of BACE2 protein was significantly higher in HRNLM cells than in HEK293 cells, while the levels of GFP were similar in HEK293 cells and HRNLM cells (Figure 1(a)). After normalization to the level of GFP, BACE2 was significantly increased to 4.75 ± 0.60-fold in HRNLM cells comparing with that in HEK293 cells (Figure 1(b)). It indicated that RCAN1 significantly upregulated BACE2 expression.

### 3.2. RCAN1 Inhibits BACE2 Turnover

**Figure 2:**
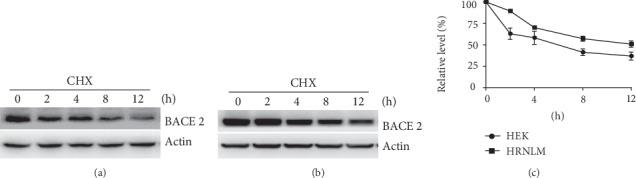
RCAN1 inhibits BACE2 turnover. (a) HEK293 cells and (b) HRNLM cells were transfected with pBACE2-mycHis. 48 hours after transfection, cells were treated with 100 *μ*g/mL CHX for 0, 2, 4, 8, or 12 h. Cell lysates were resolved by 10% SDS-PAGE. 9E10 antibody was used to detect myc-tagged BACE2 protein. *β*-Actin served as an internal control. (c) Quantification of BACE2 levels. Values represent mean ± SEM.

Protein degradation plays an important role in protein homeostasis. To determine whether RCAN1 affects BACE2 turnover rate contributing to its upregulation, the degradation rate of BACE2 was examined in HEK293 and HRNLM cells. HEK293 cells and HRNLM cells were transfected with the plasmid pBACE2-mycHis. 24 hours after transfection, cells were divided equally into five dishes. 48 hours after transfection, the cells were treated with 100 *μ*g/mL cycloheximide (CHX), a potent inhibitor of protein synthesis, to block BACE2 synthesis [16, 23, 29, 31]. The cells were harvested at 0, 2, 4, 8, and 12 h after CHX treatment, and western blot analysis was performed to determine the level of remaining BACE2 protein relative to the BACE2 level at 0 h time point. BACE2 level was decreased to 63 ± 6.6%, 58 ± 7.6%, 45 ± 6.7%, and 37 ± 4.6% at 2, 4, 8, and 12 h time points in HEK293 cells (Figures 2(a) and 2(c)). It indicated that the half-life of BACE2 was approximately 6 h in HEK293 cells. In HRNLM cells, BACE2 level was decreased to 89 ± 1.7%, 70 ± 1.6%, 55 ± 4.1%, and 51 ± 3.6% at 2, 4, 8, and 12 h time points, respectively, indicating that the half-life of BACE2 was approximately 12 h in HRNLM cells (Figures 2(b) and 2(c)). Our data indicated that RCAN1 inhibited BACE2 turnover by prolonging its half-life.

### 3.3. RCAN1 Has No Effect on Lysosome-Mediated BACE2 Degradation

**Figure 3:**
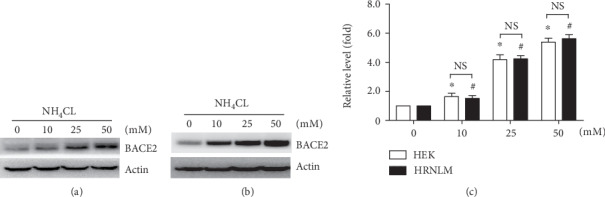
RCAN1 has no effect on lysosome-mediated BACE2 degradation. (a) HEK293 cells and (b) HRNLM cells were transfected with pBACE2-mycHis. 48 hours after transfection, cells were treated with 0, 10, 25, or 50 mM lysosomal inhibitor NH_4_Cl for 24 h. Cell lysates were resolved by 10% SDS-PAGE. 9E10 antibody was used to detect myc-tagged BACE2 protein. *β*-Actin served as an internal control. (c) Quantification of BACE2 levels. Values represent mean ± SEM; *n* ≥ 3, ∗*P* < 0.05 by two-way ANOVA.

As the previous study showed that BACE2 degradation is mediated by the lysosome pathway, the effect of RCAN1 on lysosome-mediated BACE2 degradation was examined. HEK293 cells and HRNLM cells were transfected with pBACE2-mycHis, respectively. 24 hours after transfection, cells were divided equally into four dishes. 48 hours after transfection, the lysosomal inhibitor NH_4_Cl was applied to the two cell lines. The cells were treated with 0, 10, 25, or 50 mM NH_4_Cl for 24 h. Western blot analysis showed that NH_4_Cl treatment significantly increased BACE2 levels to 1.64 ± 0.23-, 4.18 ± 0.33-, and 5.38 ± 0.27-fold, respectively, compared with those of the control in HEK293 cells, *P* < 0.05 (Figures 3(a) and 3(c)). In HRNLM cells, the level of BACE2 protein was increased to 1.52 ± 0.19-, 4.25 ± 0.22-, and 5.63 ± 0.28-fold, respectively, *P* < 0.05, when the cells were treated with 10, 25, or 50 mM NH_4_Cl (Figures 3(b) and 3(c)). However, no significant difference of relative BACE2 changes was detected between HEK293 cells and HRNLM cells when the same dose of NH_4_Cl was applied (Figures 3(a)–3(c)). It indicated that the increase of RCAN1 has no effect on lysosome-mediated BACE2 degradation.

### 3.4. RCAN1 Attenuates Proteasome-Mediated BACE2 Degradation

**Figure 4:**
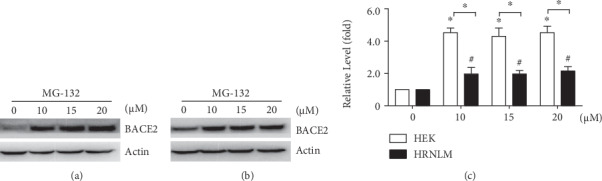
RCAN1 attenuates proteasome-mediated BACE2 degradation. (a) HEK293 cells and (b) HRNLM cells were transfected with pBACE2-mycHis. 48 hours after transfection, cells were treated with 0, 10, 15, or 20 *μ*M proteasomal inhibitor MG-132 for 12 h. Cell lysates were resolved by 10% SDS-PAGE. 9E10 antibody was used to detect myc-tagged BACE2 protein. *β*-Actin served as an internal control. (c) Quantification of BACE2 levels. Values represent mean ± SEM; *n* ≥ 3, ∗*P* < 0.05 by two-way ANOVA.

As the previous study showed that BACE2 is also degraded via the proteasome pathway, the effect of RCAN1 on proteasome-mediated BACE2 degradation was further investigated [32]. HEK293 cells and HRNLM cells were transfected with pBACE2-mycHis, respectively. 24 hours after transfection, cells were divided equally into four dishes. 48 hours after transfection, the proteasomal inhibitor MG-132 was applied to two cell lines. The cells were treated with 0, 10, 15, and 20 *μ*M MG-132 for 12 h, respectively. MG-132 treatment significantly increased BACE2 levels to 4.52 ± 0.29-, 4.30 ± 0.22-, and 4.42 ± 0.23-fold, respectively, compared with those of the control in HEK293 cells, *P* < 0.05 (Figures 4(a) and 4(c)). In HRNLM cells, the level of BACE2 protein was increased to 1.97 ± 0.23-, 1.97 ± 0.12-, and 2.15 ± 0.15-fold, respectively, *P* < 0.05, when the cells were treated with 10, 15, and 20 *μ*M MG-132 (Figures 4(b) and 4(c)). Importantly, the increase of BACE2 in HEK293 cells was much higher than that in HRNLM cells when same dose of MG-132 was applied, 4.52 ± 0.29- vs. 1.97 ± 0.23-fold at the dose of 10 *μ*M, 4.30 ± 0.22- vs. 1.97 ± 0.12-fold at the dose of 15 *μ*M, and 4.42 ± 0.23- vs. 2.15 ± 0.15-fold at the dose of 20 *μ*M, *P* < 0.05 (Figures 4(a)–4(c)). It indicated that the increase of RCAN1 attenuates proteasome-mediated BACE2 degradation.

## 4. Discussion

The dysregulation of BACE2 is observed in AD. However, the underlying mechanisms remain elusive. Our data showed that RCAN1 increases BACE2, while RCAN1 upregulation in AD is mediated by different mechanisms. It indicates that multiple stresses and risk factors of AD may implicate in the increase of BACE2 in AD, which is mediated by RCAN1 upregulation. For example, the stress hormone glucocorticoids are increased in AD, which are stimulated to be released by various stressful risk factors of AD [33]. The glucocorticoid significantly increases *RCAN1* transcription mediated by a glucocorticoid response element in the *RCAN1* promoter region [24, 34 ,35], indicating that stress hormone may contribute to the BACE2 upregulation. In addition, *ApoE* allele*4*, a known risk factor of AD, markedly increases RCAN1 expression, indicating that it may also promote BACE2 expression [36]. NF-*κ*B promotes *RCAN1* transcription, indicating that inflammation may play a key role in the upregulation of RCAN1 and subsequent BACE2 upregulation in AD [37]. The putative transcription factor HIF-1 binding site within the *RCAN1* promoter region may mediate the alteration of RCAN1 expression under hypoxia, ischemia, and stroke conditions, promoting AD development. It is also supported by the fact that hypoxia, ischemia, and stroke are also risk factors of AD. Moreover, chronic hyperglycemia, the characteristic of diabetes, increases RCAN1 expression in pancreatic *β* cells, suggesting that chronic hyperglycemia may also contribute to RCAN1 elevation in the brain [38]. As both stroke and diabetes are age-associated diseases, their role in RCAN1 regulation may also be affected by the aging process [39]. The above evidence suggests that stroke and diabetes may promote the upregulation of BACE2 mediated by increased RCAN1 expression. In fact, inhibition of BACE2 counteracts IAPP-induced *β* cell loss and functional defects [40, 41]. Furthermore, calcineurin and GSK3*β* activities are significantly altered in AD brains, which are involved in regulating RCAN1 degradation rate by modulating its phosphorylation status [42–48]. It indicates that the dysregulation of calcineurin and GSK3*β* may be implicated in the increase of BACE2 in AD, which is mediated by RCAN1 upregulation, although further investigation is needed.

Our data showed that RCAN1-induced increase of BACE2 expression is caused by attenuating proteasome-mediated BACE2 degradation. Although the mechanism of RCAN1 attenuating proteasome-mediated BACE2 degradation is not clear, the following mechanisms may be implicated in RCAN1 attenuating the proteasome-mediated BACE2 degradation. Previous studies showed that RCAN1.1S could stabilize oligosaccharyltransferase (OST) by interacting with the OST component ribophorin I (RPN I) [49]. It is possible that RCAN1 may stabilize BACE2 by directly interacting with BACE2. In addition, RCAN1.1S promotes the N-glycosylation of BACE1 and BACE2, a type of protein posttranslational modification, which is associated with their upregulation [49]. It is possible that RCAN1 may also facilitate BACE2 N-glycosylation contributing to the increase of BACE2 expression. Moreover, RCAN1 not only regulates the activity of various transcriptional factors, e.g., NFAT, but also shows RNA binding activity, which may be implicated in the increase of BACE2 by regulating the expression of OST components or ubiquitin-proteasome system- (UPS-) associated molecules.

## 5. Conclusions

In conclusion, our work indicates that RCAN1 inhibits BACE2 turnover by attenuating proteasome-mediated BACE2 degradation, leading to the upregulation of BACE2. It advances our understanding of BACE2 regulation and provides a potential mechanism of BACE2 dysregulation in AD. Moreover, it provides a novel insight that RCAN1 dysregulation promotes the pathogenesis of AD through BACE2 7in addition to other known mechanisms. It suggests that RCAN1 and BACE2 may be potential targets for AD treatment.

## Data Availability

The data used to support the findings of this study are available from the corresponding author upon request.
